# Editorial

**Published:** 1841-08-07

**Authors:** 


PHILADELPHIA, AUGUST 7, 1841.

### PENNSYLVANIA STATE ASYLUM FOR THE INSANE.

A long time since we ventured to lend our aid to the exertions of some philanthropic citizens to procure the erection of a public insane asylum. The insane poor of Pennsylvania were heretofore almost every where without adequate accommodation, and without any at all in most places. Our courts had no means of keeping in custody the vicious insane, who had been acquitted from the penalties of our criminal courts, because their insanity had been established, and society was without the means of self-protection against the unrestrained maniac, who to-day might set a house on fire, and to-morrow murder a fellow creature. Humanity and self-interest were thus powerfully appealed to, and the state of Pennsylvania has at length followed the example of other states who had preceded her in this respect. At the last session of the Legislature, $120,000 was given for the purpose of establishing a state insane asylum.

By this law it is provided,

*Section* 6. That the government of said asylum shall be vested in nine trustees, to be appointed by the Governor, who shall serve without compensation : of those first appointed, three shall serve for one year, three for two years, and three for three years : and at the expiration of the respective periods, the vacancies to be filled by appointments for three years; and should any vacancy occur by the death, resignation, or otherwise, of any trustee, such vacancy shall be filled by an appointment for the unexpired time of such trustee. The said trustees shall have charge of the general interests of the institution ; they shall appoint the superintendent, who shall be a skilful physician, and shall always reside at the asylum ; and they shall make such by-laws and regulations as they may think necessary ; they shall also appoint a treasurer, who shall be approved by the Governor, and give sufficient bonds to the commonwealth for the faithful discharge of his duties; and they shall appoint such other officers and assistants, and fix upon the compensation for their services, as may be necessary for the efficient and economical administration of the affairs of the institution. Said trustees shall have power to take and hold in trust for the use and benefit of said asylum, any grant or devise of land, and any donation or bequest of money or other personal property, to be applied to the maintenance of insane persons in or to the general use of the asylum.

*Section* 8. That the proper courts of this commonwealth shall have power to commit to said asylum, any person who, having been charged with an offence punishable by imprisonment or death, shall have been declared by the verdict of a jury or otherwise, to the satisfaction of the court, to have been insane at the time the offence was committed, and who still continues insane.

*Section* 9. That if any person shall apply to any court of record within this commonwealth, having final jurisdiction of offences which are punishable by imprisonment for the term of ninety days or longer, for the commitment to said asylum of any insane person within the county in which such court has jurisdiction, it shall be the duty of said court to inquire into the fact of insanity, and if such court shall be satisfied that such person is, by reason of insanity, unsafe to be at large, or is suffering any unnecessary duress or hardship, such court shall, on the application aforesaid, commit such insane person to said asylum.

*Section* 11. The several constituted authorities having care and charge of the poor in the respective counties, districts, and townships of this commonwealth, shall have authority to send to the asylum such insane paupers under their charge as they may deem suitable subjects for its treatment; and they shall be severally chargeable with the expense of the care and maintenance, and removal to and from the asylum of such paupers.

*Section* 12. That in the admission of paupers to said asylum, preference shall always be given by the trustees to cases in which the disease is of recent origin ; and if the trustees shall in any case deem it for the interest of the asylum, or of the patient, that he or she should be removed, the superintendent shall give notice to those who are responsible for his or her support, and, if he or she shall not be removed within thirty days after such notice, the trustees may cause him or her to be removed at the expense of the person or persons, body corporate or politic, who may be liable for his or her support.

The Governor has appointed John K. Kane, George Rundle, and John W. Ashmead commissioners for building the asylum, and the following gentlemen trustees :—Richard Rush, Dr. Geo. McClellan, John White, for one year. Isaac Collins, Michael W. Ash, C. Wallace Brooke, for two years. Jacob Lex, Dr. R. Dunglison, James Campbell, for three years.

We sincerely hope that the institution will be so conducted as to carry into the most effectual execution the benevolent objects of the law, and of the munificent appropriation of the state, and, at the same time, to further the interests of science and the cause of general philanthropy.

